# Macrophage Depletion Improves Endothelial Insulin Resistance and Protects against Cardiovascular Injury in Salt-Sensitive Hypertension

**DOI:** 10.1155/2020/5073762

**Published:** 2020-08-11

**Authors:** Yue-Yang Liu, Jun Luo, Ruiping Cai, Junjie Zhang, Qian Xu, Yuantong Tian, Ming-Sheng Zhou

**Affiliations:** ^1^Department of Physiology, Shenyang Medical University, Shenyang 110034, China; ^2^Department of Cardiology, The Affiliated Ganzhou Hospital of Nanchang University, Ganzhou 341000, China; ^3^The Open Project of Key Laboratory of Prevention and Treatment of Cardiovascular and Cerebrovascular Diseases, Ministry of Education, Gannan Medical University, Ganzhou 341000, China

## Abstract

Vascular endothelial insulin signaling is critical for the maintenance of vascular and metabolic homeostasis. We have previously shown that in hypertensive Dahl rats, impaired vascular insulin action is linked to angiotensin II activation of the NF*κ*B inflammatory pathway. Macrophage polarization (M1) has implicated in hypertensive and metabolic diseases. Here, we investigated the effect of macrophage depletion using liposome-encapsulated clodronate (LEC) on endothelial insulin resistance and cardiovascular remodeling in Dahl salt-sensitive (DS) rats. High salt intake (HS) for 5 weeks increased systolic blood pressure (SBP: 192 ± 5 vs. 144 ± 4 mmHg in NS, *p* < 0.05), aortic and cardiac hypertrophy, cardiac fibrosis, and impaired acetylcholine- and insulin-induced vasorelaxation, accompanied by impaired insulin activation of endothelial nitric oxide synthases (eNOS)/NO signaling. HS rats had a significant increase in CD68 (a monocyte/macrophage marker) expression in the aorta and the heart. LEC reduced SBP (168 ± 5 mmHg, *p* < 0.05) and cardiovascular injury and improved acetylcholine- and insulin-mediated vasorelaxation and insulin signaling molecules with a reduction in the macrophage infiltration in the aorta and the heart. HS rats also manifested an increase in the aortic expressions of inflammatory cytokines, including the ratio of phosphorylated inhibitory kappa B (I*κ*b)/I*κ*b, tumor necrosis factor *α*, and phosphorylated c-Jun N-terminal kinase (JNK) and oxidative stress, which were reduced in HS/LEC rats. Our results suggest that in salt-sensitive hypertension, macrophage may importantly contribute to endothelial insulin resistance, vascular inflammation, and injury. These findings support the idea that macrophages may be a new target for immunotherapy of vasculopathy in hypertensive and metabolic disorders.

## 1. Introduction

Impaired insulin signaling is the central feature of the metabolic syndrome (MS); insulin resistance is often associated with hypertension, especially salt-sensitive hypertension; this association may promote cardiovascular (CV) diseases and microvascular complication in type II diabetes mellitus [1, 2]. Increasing evidences have shown that insulin resistance exists not only in traditional insulin metabolic organs but also in the heart and vessels [3]. The vascular action of insulin is to activate the phosphoinositide kinase (PI3K)/endothelial nitric oxide synthase (eNOS) pathway in the endothelium to induce vascular relaxation; insulin stimulation of this pathway may also importantly contribute to glucose homeostasis via regulation of blood flow in the skeletal muscle [4]. Endothelial insulin resistance is characterized by a selective impairment of insulin activation of the PI3K/NO pathway in endothelium [5]. On the other side, insulin activates the mitogen-activated protein kinase (MAPK) pathway, which may promote hypertension, vascular hypertrophy, and CV remodeling. Therefore, impaired endothelial insulin signaling may be an important link among hypertension, CV, and metabolic diseases [4, 6, 7].

Both hypertension and insulin resistance are associated with chronic low-grade inflammation [8, 9]. It has shown that immune cells, especially monocytes/macrophages, are recruited in the vascular walls of various hypertensive animals [10, 11]. The inflammatory cytokines released by monocytes/macrophages have important vascular biological effects, such as the impairment of endothelial function, the promotion of vascular inflammation, cardiac and vascular fibrosis, and hypertrophy [12]. Several studies have shown that macrophage depletion can preserve endothelial function and reduce oxidative stress and cardiovascular and renal injury in various hypertensive animals [10, 13, 14].

Dahl salt-sensitive (DS) rat is a paradigm of salt-sensitive hypertension in humans. We have previously shown that hypertensive DS rat is a vascular phenotype which is characterized by impairment of endothelial function, vascular inflammation, and vascular dysfunctional and structural injury associated with metabolic and vascular insulin resistance [15–17]. The impairment of endothelial function and vascular insulin signaling is mechanistically linked to angiotensin (Ang) II activation of the nuclear factor (NF) *κ*B/tumor necrosis factor (TNF) *α* inflammatory pathway [17]. It has been shown that macrophages play an important role in hypertensive end-organ damage [11, 12]; macrophages are also an important source to produce reactive oxygen species (ROS) and inflammatory cytokines, such as TNF*α*, which may inhibit insulin signaling and induce vascular inflammation [8, 18]. In the present study, we investigated the role of macrophages in endothelial insulin resistance and CV remodeling, using chemical depletion of monocyte/macrophage with the administration of liposome-encapsulated clodronate (LEC) in DS rats.

## 2. Methods

### 2.1. Animals and Experimental Protocols

Six-week-old male inbred DS rats were purchased from Beijing Charles River Animal Laboratory (Beijing, China) and housed in the animal care facility with a 12 : 12 h light-dark cycle. The rats were fed a standard rat chow diet and adapted to a new environment for two weeks. All animal protocols were conformed to the international standards stated in the Guide for the Care and Use of Laboratory Animals and approved by the Institutional Animal Care and Use Committee of Shenyang Medical University. DS rats were randomly divided into the following three groups and treated for 5 weeks: (1) normal salt control (NS, 0.5% NaCl, *n* = 8) diet with PBS-liposome treatment, (2) high salt (HS, 4% NaCl, *n* = 8) diet with PBS-liposome treatment, and (3) HS diet with LEC treatment (HS/LEC, *n* = 8). LEC is a chemical compound which is widely used *in vivo* for the monocytes/macrophage depletion [19]. LEC at the HS/LEC group was administrated through the tail vein injection at the dose of 20 mg/kg body weight. LEC was given the day before the rats received HS treatment and repeated every three days thereafter until the end of the experiments. The rats in the NS or HS control group were injected with the same volume of PBS-liposome. The depletion of monocytes by LEC was confirmed by blood smears with Giemsa staining that showed that the number of monocytes in the peripheral blood in HS/LEC rats was reduced by 60-70%; the results were consistent with the literatures [10, 19]. Briefly, a small amount of blood (about 1-2 drops) on the 2^nd^ day after LEC treatment was taken for blood smears, and Giemsa staining was performed. At least a total of 350 white blood cells per slide were counted by a blind observer; the monocytes, granulocytes, and lymphocytes were identified according to their nuclear morphology; the number and percentage of various white blood cells were calculated. Systolic blood pressure (SBP) was measured in a quiet and dark room in conscious rats, at baseline and once a week after treatment until the end of the experiment, using the tail-cuff method (Softron Biotech Inc., Beijing). The rats were trained daily for 5 consecutive days for blood pressure measurement before the experiment was performed; at least five successive measurements were recorded and averaged as a value of blood pressure measurement. At the end of the study, after anesthetizing with an overdose of sodium pentobarbital (100 mg/kg I.P), the rats were euthanized by decapitation; the heart and the aorta were harvested. The heart and aortas (from the arch of the thoracic aorta to the origin of the mesenteric artery from the abdominal aorta) were weighted and used as the indices of cardiac and aortic hypertrophy, respectively.

### 2.2. Histological Study

Two micrometers of the thoracic aorta and one piece of left ventricular tissue were fixed in 4% paraformaldehyde in phosphate-buffered saline. The tissues were embedded in paraffin and cut a section thickness of 4 *μ*m. After the sections were deparaffined, hematoxylin and eosin staining was performed for the evaluation of aortic and cardiomyocyte hypertrophy. Four images in four nonconsecutive slides per sample from the aortic section were acquired with an Olympus DS-41 microscope equipped with a DP-72 camera and analyzed with the quantitative digital image analysis system, and the aortic wall thickness from three different levels at the top, middle, and lower lines of the image was measured. The aortic wall thickness was measured from the intima to the media but the exclusion of the adventitia, because some arterial preparations may not have a clear boundary of the adventitia. The average cross-sectional area of cardiomyocytes was measured using the quantitative digital image analysis system (Media Cybernetics, Rockville); at least 100 cardiomyocytes in four randomly selected fields per slide were measured and averaged as a single measurement. The left ventricle section was also stained with Masson's trichrome (Sigma-Aldrich, St. Louis, MO) to assess the myocardial fibrosis. A semiquantitative analysis was used to assess the myocardial collagen content, using the Image-Pro Plus image analysis system. The results were expressed as the percentage of positive collagen-stained areas with total areas of selected cardiac tissue. All histologic examination, image quantitation, and representative photomicrographs were taken in a blind fashion without the knowledge of the experimental group.

### 2.3. Vascular Reactivity Study

Endothelium-dependent relaxation to acetylcholine (Sigma-Aldrich, St. Louis, MO) or insulin (Sigma-Aldrich, St. Louis, MO) in the aortic rings was determined using an organ chamber bath (DMT Inc., Denmark), as previously described [17]. Briefly, the aorta was cleared of adherent connective tissues and cut into 3 mm rings. After resting tension (1 g) was equilibrated for one hour, the aortic rings were exposed to PSS solution containing 60 mmol/L KCl twice at intervals of 30 minutes to induce vasoconstriction response. The aortic rings were precontracted with 70% of maximal norepinephrine-induced vasoconstriction (about 30 nmol/L norepinephrine); then, an accumulative dose of acetylcholine (10^−9^ to 10^−5^ mol/L) or insulin (10^−9^ to 10^−6^ mol/L) was added in the organ chamber; endothelium-dependent relaxation to acetylcholine or insulin was measured. In some experiments, the aortic rings were preincubated with L-NAME (100 *μ*mol/L, a nonspecific nitric oxidase synthase inhibitor) for 30 minutes to inhibit NOS activity; insulin-induced vasorelaxation was examined after the blockade of NO production. Acetylcholine- or insulin-induced vasorelaxation was expressed as percentage inhibition of norepinephrine-induced vasoconstriction. An agonist-induced maximal vascular relaxation (*E*_max_) and the concentration of an agonist required for the induction of a half-maximal response (ED_50_) were calculated from the concentration-response curve, using the best fit to a logistic sigmoid function.

### 2.4. Immunohistochemistry and Immunofluorescence Staining for Aortic CD68 Expression

One piece of the left ventricle or thoracic aortic tissues was embedded in paraffin and cut into 4 *μ*m thick section. After the deparaffinization and hydration, the slides were placed on the microwave for one hour at 60°C for antigen retrieval. The slides were incubated with primary antibody against CD68, a monocyte/macrophage lineage marker (1 : 100 dilutions with TBST buffer, Santa Cruz Technology.), overnight at 4°C. For immunohistochemistry, the secondary antibody was biotinylated horse anti-mouse IgG (Vector Laboratories). A Vectastain Elite ABC Kit (Vector Laboratories) was used according to the manufacturer's protocol. The nuclei were visualized by counterstaining with DAPI. The images were digitized and semiquantified using the Image-Pro Plus version 6.0 software system. Percentage of the CD68+ area was calculated by dividing the total selected area; the data was expressed as a fold change vs. the control group. For immunofluorescence, the sections were incubated with Cyanine 3- (CY3-) conjugated goat anti-mouse IgG (Thermo Fisher Scientific) at room temperature for one hour. The nuclei were stained with DAPI. Monocytes/macrophages (CD68-positive cells) in the aortic wall were viewed using a fluorescence microscope, and the CD68-positive cells were counted by an experienced reviewer who was blind to experimental groups. At least 5 images per section were examined; the number of positive cells per image was expressed by per mm^2^ area of the image. The section without primary antibody was used as a negative control.

### 2.5. Determination of In Situ Superoxide Anion (O_2_^−^) Production

The oxidative fluorescent dihydroethidine (DHE, Sigma-Aldrich, St Louis, MO) was used to determine in situ O_2_^−^ production in the aortic rings as previously described [15]. Briefly, the fresh aorta was embedded in OCT compound, snap-frozen in liquid nitrogen; 5 *μ*m thick section was cut and mounted in the slide. The slides were incubated with 2 *μ*mol/L DHE in HEPES buffer at 4°C for 30 minutes. The images were taken within 30 minutes after DHE staining to avoid fluorescent degradation, using a Leica confocal fluorescence microscope (Leica Microsystems Inc., Mannheim, German). The oxidative fluorescence intensity of images was measured with double blind; the reviewers do not know which experimental group the slides belong to; the average fluorescent intensities were calculated by total fluorescent intensities divided by total selected area.

### 2.6. Western Blot

The aortas were homogenized with a 3-time volume of lysis buffer containing protease inhibitors aprotinin (10 *μ*g/mL), leupeptin (10 *μ*g/mL), and PMSF (1 mmol/L). The lysates were centrifuged at 12000 g for 30 minutes. The supernatants were used for the determination of protein concentration by the Bio-Rad protein assay. Thirty *μ*g of proteins of the supernatants was loaded with SDS-PAGE gel; after electrophoresis, the separated proteins were transferred to a nitrocellulose membrane. The membrane was incubated with specific primary antibodies at 4°C overnight; the primary antibodies include anti-TNF*α*, inhibitory *κ*B (I*κ*B), pI*κ*B (Santa Cruz Biotech.) and phosphor-insulin receptor substrate 1 (pIRS1 Ser612), c-Jun N-terminal kinase (JNK), and pJNK (cell signaling). After washing with TBS, the membranes were incubated with appropriate horseradish peroxidase-conjugated secondary antibodies at room temperature for one hour. The signals were detected by luminal-enhanced chemiluminescence ECL reagents (Thermo Fisher Scientific, MA) and quantified by densitometric analysis with Image J. The data was normalized to *β*-actin (Santa Cruz Biotech.) and expressed as a fold increase versus the control group. The phosphor-I*κ*B or phosphor-JNK was normalized with a total of I*κ*B or JNK.

### 2.7. Insulin-Stimulated Akt and eNOS Phosphorylation

The aorta was cut into small rings. The aortic rings were incubated with 100 nmol/L insulin (Sigma-Aldrich Inc.) in DMEM medium bubbled with 95% at 37°C for 30 minutes to stimulate the insulin pathway. The protein expression of insulin-stimulated pAkt (Ser473) or peNOS (Ser 1177) was determined by Western blot analysis, using the specific primary antibodies against pAkt (Ser473) or peNOS (Ser117) (cell signaling), as described above.

### 2.8. Data Analysis

All experimental results were expressed as mean ± standard error of the mean (SEM). Statistical analyses were performed using SPSS 18.0 software (SPSS Inc., Chicago, IL); the statistical significance of difference was determined by one-way or two-way ANOVA followed by the Bonferroni corrections for multiple comparisons between groups. The values were considered significant when *p* < 0.05.

## 3. Results

### 3.1. LEC Lowered SBP, Circulating Monocyte Population and Cardiac and Aortic CD68 Expression in Hypertensive DS Rats

**Figure 1:**
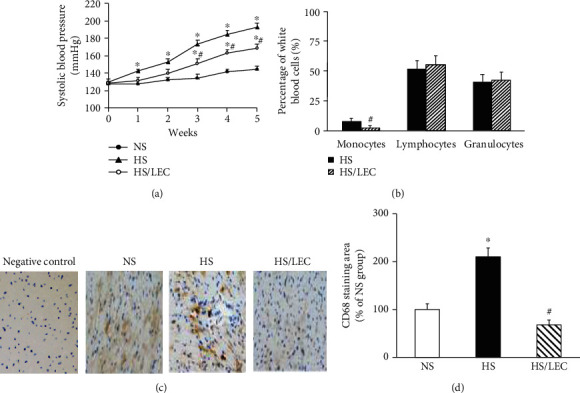
Effects of liposome-encapsulated clodronate (LEC) treatment on systolic blood pressure (SBP, a), the distributions of leukocytes (b), and cardiac CD68 expression (c, d) in hypertensive DS rats. The representative images of CD68 expression in the heart assessed by immunohistochemistry (c); quantitative assessment of CD68 expression (d). NS: the rats fed a normal salt diet (0.5% NaCl); HS: the rats fed a high salt diet (4% NaCl); HS/LEC: the rats treated with HS plus LEC. The section incubated with IgG without a primary antibody was used as a negative control. Statistical comparisons were performed by one-way (d) and two-way (a) ANOVA followed by the Bonferroni correction for multiple comparisons between groups, and Student's *t* test (b). All data were expressed as mean ± SEM. *N* = 8, ^∗^*p* < 0.05 vs. NS group, ^#^*p* < 0.05 vs. HS group.

DS rats in the HS group had a significant increase in SBP (192 ± 5 vs. 144 ± 4 mmHg in the NS group, *p* < 0.05). LEC treatment significantly lowered SBP in HS rats (168 ± 5 vs. 192 ± 5 mmHg in the HS group, *p* < 0.05, Figure 1(a)). There was no significant difference in heart rate and fasting plasma level of glucose among the NS, HS, and HS/LEC rats (Table 1). Hypertensive DS rats have a tendency to reduce body weight but did not reach statistical significance (*p* = 0.08) as compared with NS rats; LEC treatment did not affect the gain of body weight in HS rats (Table 1). LEC is a drug which can effectively and selectively deplete monocyte/macrophage lineage, as LEC can be engulfed by macrophage to induce monocyte/macrophage apoptosis [19]. In the present study, we used the same dosage of LEC to treat the rats as previously reported [10, 13]; LEC treatment reduced the numbers of monocytes in the peripheral blood by 67% without significant changes in the population of lymphocyte or granulocyte lineage in hypertensive DS rats (Figure 1(b)).

**Figure 2:**
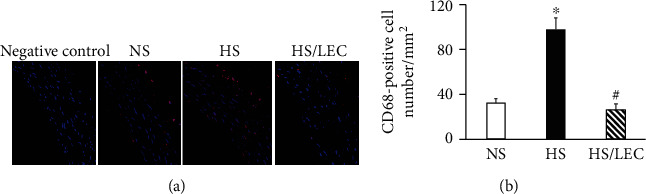
LEC significantly reduced CD68-positive cell number in the aorta of hypertensive Dahl rats. (a) Representative images of CD68-positive cells (red), determined by immunofluorescence. (b) Quantitative analysis of CD68-positive cell number. Statistical comparisons were performed by one-way (a) ANOVA followed by the Bonferroni correction for multiple comparisons between groups. *N* = 8, *p* < 0.05 vs. NS group, *p* < 0.05 vs. HS group.

Immunohistochemistry staining showed that the CD68-positive staining area was significantly increased in the heart tissue of hypertensive DS rats, which was reduced in the HS/LEC rats (Figures 1(c) and 1(d)). Immunofluorescence staining showed that CD68 expression was markedly increased in the aortic wall of HS rats, especially in the adventitia of the aorta. LEC significantly reduced CD68 expression in HS rats (Figure 2). These results indicate that increased macrophage infiltration occurs in the cardiovascular system of hypertensive DS rats, and LEC has a selective target on the monocyte/macrophage lineage with the reduction in circulating monocytes and tissue macrophages.

### 3.2. LEC Attenuated Aortic and Cardiac Hypertrophy and Cardiac Fibrosis in Hypertensive DS Rats

**Figure 3:**
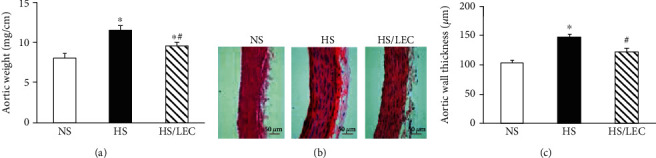
The depletion of macrophage by LEC attenuated aortic hypertrophy in hypertensive DS rats. Aortic hypertrophy was assessed by aortic weight (a) and aortic wall thickness (b, c). The representative images of aortic wall thickness stained with hematoxylin and eosin (b). Quantitative analysis of aortic wall thickness (c). Statistical comparisons were performed by one-way (a, c) ANOVA followed by the Bonferroni correction for multiple comparisons between groups. *N* = 8, ^∗^*p* < 0.05 vs. NS group, ^#^*p* < 0.05 vs. HS group.

**Figure 4:**
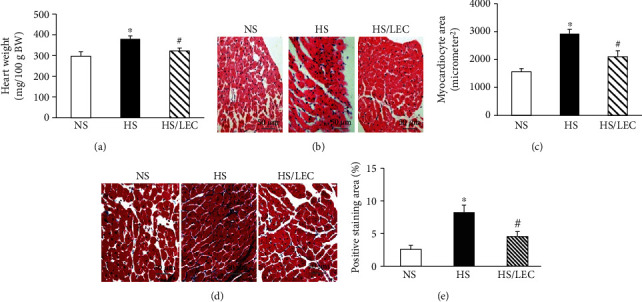
The depletion of macrophage by LEC attenuated cardiac hypertrophy (a–c) and cardiac fibrosis (d, e) in hypertensive DS rats. The cardiac hypertrophy was assessed by heart weight (a) and cardiomyocyte sectional area (b, c). The representative images of cardiomyocyte sectional area stained with hematoxylin and eosin (b), the quantitative analysis of cardiomyocyte sectional area (c), the representative images of heart section stained with Masson's trichrome for evaluation of cardiac fibrosis (d), and the quantitative assessment of the positive collagen-staining area in the heart (e). Statistical comparisons were performed by one-way ANOVA followed by the Bonferroni correction for multiple comparisons between groups. *N* = 8, ^∗^*p* < 0.05 vs. NS group, ^#^*p* < 0.05 vs. HS group.

Hypertensive DS rats had higher aortic weight (11.4 ± 0.6 vs. 8.0 ± 0.5 mg/cm in the NS group, *p* < 0.05) and heart weight (380 ± 15 vs. 285 ± 21 mg/100 g BW in NS rats, *p* < 0.05); LEC treatment significantly lowered the aortic weight (9.5 ± 0.5 vs. 11.4 ± 0.6 mg/cm in the HS group, *p* < 0.05, Figure 3(a)) and heart weight (322 ± 14 vs. 380 ± 15 mg/100 g BW in HS rats, *p* < 0.05, Figure 4(a)) in hypertensive rats. In addition, we also normalized the aortic weight by body weight (AW/BW); the ratio of AW/BW in HS rats was significantly higher than that in NS rats, which was reduced in HS/LEC rats (Table 1). HE staining revealed that aortic wall thickness and medial growth were significantly increased in the HS rats, which was significantly reduced in the HS/LEC rats (Figures 3(b) and 3(c)). Cardiomyocyte sectional area was also significantly increased in hypertensive DS rats, which was reduced in the HS/LEC rats (*p* < 0.05, Figures 4(b) and 4(c)). Masson's trichrome staining showed that hypertensive HS rats had a more positive collagen-stained area of myocardial tissue than the NS rats. LEC treatment significantly reduced the positive collagen-stained area in hypertensive HS rats (Figures 4(d) and 4(e)).

### 3.3. LEC Improved Insulin-Induced Vasorelaxation and Vascular Insulin PI3K/Akt Signaling Molecule

**Figure 5:**
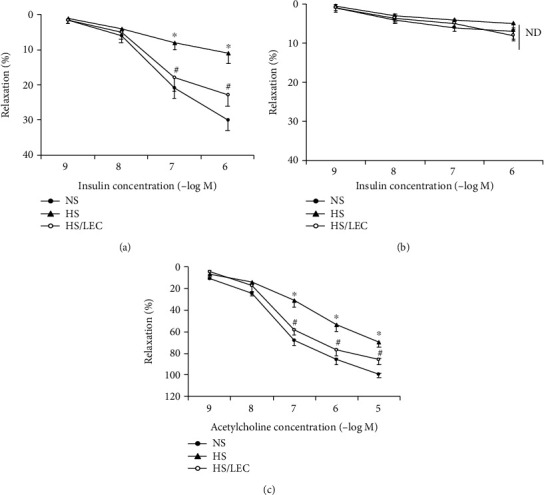
The macrophage depletion by LEC improved endothelium-dependent relaxation to insulin (a, b) and acetylcholine (c) in hypertensive DS rats. (a) Insulin-induced vasorelaxation in the absence of NOS inhibitor L-NAME; (b) insulin-induced vasorelaxation in the presence of L-NAME. Statistical comparisons were performed by two-way ANOVA followed by the Bonferroni correction for multiple comparisons between groups. ND: no significant difference. *N* = 8, ^∗^*p* < 0.05 vs. NS group, ^#^*p* < 0.05 vs. HS group.

**Figure 6:**
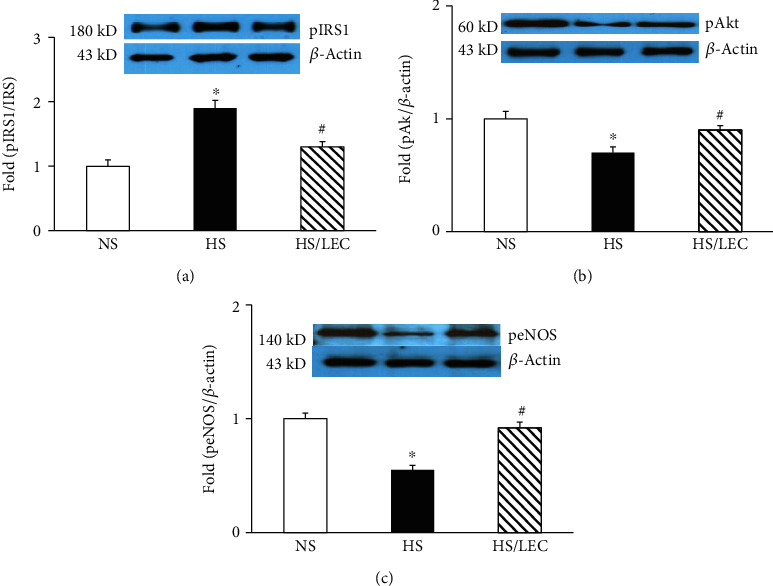
The macrophage depletion by LEC improved vascular insulin signaling through IRS1/Akt/eNOS in hypertensive DS rats. The aortic tissues were incubated with insulin (100 nmol/L) for 30 minutes to stimulate insulin signaling. The protein expression of phosphor-IRS1 (Ser 612, a), phosphor-Akt (Tyr 473, b), and phosphor-eNOS (Ser 1177, c) was assessed by Western blot. Statistical comparisons were performed by one-way ANOVA followed by the Bonferroni correction for multiple comparisons between groups. *N* = 6, ^∗^*p* < 0.05 vs. NS group, ^#^*p* < 0.05 vs. HS group.

Vascular response to KCl (60 mmol/L) was comparable among NS, HS, and HS/LEC (Table 1). Insulin-induced vasorelaxation was impaired in hypertensive DS rats (*E*_max_: 11 ± 3 vs. 30 ± 3% in NS rats, *p* < 0.05; ED50: 7.5 ± 0.1 vs. 7.4 ± 0.1 –log molar in NS rats, *p* > 0.05); LEC treatment significantly improved insulin-induced vasorelaxation in the HS rats (*E*_max_: 23 ± 3 vs. 11 ± 3% in HS rats, *p* < 0.05; ED50: 7.5 ± 0.2 vs. 7.4 ± 0.1 –log molar in HS rats, *p* > 0.05, Figure 5(a)). Moreover, preincubation with NOS inhibitor L-NAME almost abolished insulin-induced vasorelaxation in all groups of rats (Figure 5(b)). Endothelium-dependent relaxation to acetylcholine was also impaired in the aorta of hypertensive DS rats. LEC treatment significantly improved acetylcholine-induced vasorelaxation (Figure 5(c)). It is well known that insulin induces vasorelaxation via the activation of the IRS1/Akt/eNOS pathway in the endothelium. There was no significant difference in the protein expression of total IRS1 among all three groups of rats. However, the aortic expressions of the phosphor-IRS1 (Ser612) and the ratio of phosphor-IRS1 (Ser612)/total IRS1 were significantly increased in hypertensive DS rats, which were reduced in the HS/LEC rats (Figure 6(a)). It has shown that phosphor-IRS1 at serine residue can inhibit insulin activation of downstream molecules, such as Akt and eNOS phosphorylation [20]. The vascular expressions of pAkt (Ser473) and peNOS (Ser1177) were significantly reduced in the HS rats and restored in the HS/LEC rats (Figures 6(b) and 6(c)).

### 3.4. LEC Reduced the Expressions of Proinflammatory Cytokines and Oxidative Stress in the Aorta of Hypertensive DS Rat

**Figure 7:**
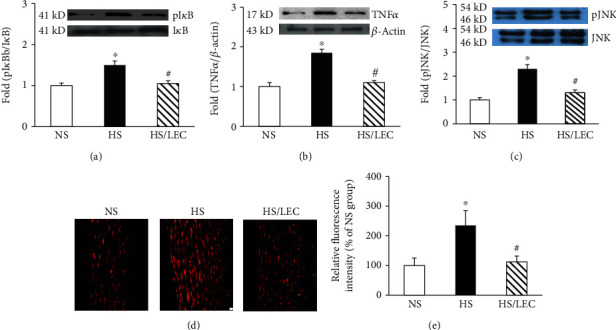
The macrophage depletion by LEC reduced the ratio of phosphor-I*κ*B/I*κ*B (a), TNF*α* (b) and phosphor-JNK expression (c), and oxidative fluorescence intensity (d, e) in the aorta of hypertensive DS rats. The representative images of aortic sections stained with DHE for the evaluation of oxidative fluorescence intensity using a confocal fluorescence microscope (d); the quantification of average fluorescence intensities (e). Statistical comparisons were performed by one-way ANOVA followed by the Bonferroni correction for multiple comparisons between groups. *N* = 6‐8, ^∗^*p* < 0.05 vs. NS group, ^#^*p* < 0.05 vs. HS group.

In unstimulated cells, NF*κ*B is associated with inhibitory *κ*B (I*κ*B); NF*κ*B is activated by I*κ*B degradation which is caused by I*κ*B phosphorylation. Increased phosphor-I*κ*B or the ratio of phosphor-I*κ*B/I*κ*B is an index for NF*κ*B activation [21, 22]. The aortic expression of phosphor-I*κ*B*α* (Ser312) and the ratio of phosphor-I*κ*B*α*/I*κ*B*α* were significantly increased in the hypertensive DS rats. LEC treatment restored the expression of phosphor-Ser^312^ I*κ*B*α* and the ratio of phosphor-Ser^312^ I*κ*B*α*/I*κ*B*α* (Figure 7(a)). TNF*α* is one of the proinflammatory cytokines which is mainly produced by the activated macrophages. The expression of TNF*α* was significantly increased in the aorta of hypertensive DS rats; LEC treatment lowered TNF*α* expression in the HS rats (Figure 7(b)). JNK is one of the major inflammatory cytokines to inhibit insulin signaling via the induction of IRS1 phosphorylation at serine residues in hypertensive and metabolic diseases [20]. As shown in Figure 7(c), there was no significant difference in total JNK expression among NS, HS, and HS/LEC rats; however, pJNK (Ser186) was markedly increased in hypertensive HS rats, and LEC treatment significantly reduced pJNK expression in the HS rats.

The macrophages are major producers of ROS in the infiltrating tissues [23, 24]. NADPH oxidase [25] is a major enzyme to generate ROS in the vasculature, heart, and phagocytes. We determined O_2_^−^ production in the aorta by a confocal fluorescence microscope using oxidative fluorescent dihydroethidine. As shown in Figures 7(d) and 7(e), oxidative fluorescence intensities were significantly increased in the aorta of hypertensive DS rats, which were significantly reduced in DS/LEC rats.

## 4. Discussion

Vascular endothelial insulin signaling is critical for the maintenance of vascular and metabolic homeostasis. The impairment of vascular insulin signaling has implicated in the development of macro- and microvascular diseases in hypertensive and metabolic diseases [6, 17]. We have previously shown that the impairment of endothelial insulin NO signaling is associated with Ang II activation of the NF*κ*B inflammatory pathway in hypertensive DS rats [16, 17]. The present study demonstrates for the first time that the chemical depletion of macrophages by LEC improves insulin-induced vasorelaxation and insulin stimulation of eNOS/NO signaling in hypertensive rats. Moreover, the depletion of macrophage by LEC also improves endothelium-dependent relaxation to acetylcholine and reduces vascular inflammation, oxidative stress, and cardiovascular remodeling [16]. These results suggest that macrophage activation may play an important role in the impairment of vascular insulin signaling and cardiovascular injury in hypertension, especially in salt-sensitive hypertension.

It has been shown that chronic inflammation plays a critical role in the pathogenesis of hypertensive and metabolic diseases [12, 26–28]. Hypertension and insulin resistance are two important components in the MS, which may represent an example of immunometabolic vascular disease [12, 29]. The recruitments of immune cells, such as monocytes/macrophages, in the vascular walls or visceral adipose tissues have been reported in various hypertensive animal models [30–32]. The depletion of macrophages using either chemicals or genetic manipulation has shown to reduce blood pressure and improve endothelial function and cardiac and vascular remodeling in hypertensive animals [11, 30], but it is still unclear whether macrophages play a direct role in the impairment of vascular insulin action and insulin signaling in hypertensive or metabolic diseases. Vascular insulin signaling plays two important roles: the induction of vasorelaxation via the regulation of endothelium NO synthesis/release, and the enhancement of glucose disposal via the regulation of blood flow in the skeletal muscle [6]. Thus, the impairment of vascular insulin signaling may represent a specific vascular phenotype which is associated with vascular and metabolic dysfunction. We showed that expressions of macrophage marker CD68 were significantly increased in the vasculature and the heart of hypertensive rats; LEC specifically targeted monocyte/macrophage lineage and reduced tissue macrophage infiltration; macrophage depletion not only reduced blood pressure and cardiovascular injury but also improved insulin-mediated vasorelaxation and insulin PI3K/NO pathway, suggesting the important role of macrophages in endothelial insulin signaling and the vascular-metabolic dysfunction.

Macrophages may change their phenotype and function in response to various environmental signals [33, 34]. Macrophage-released inflammatory cytokines may initiate vascular inflammation and endothelial dysfunction [35]. TNF*α* is one of the best described inflammatory cytokines in disturbed insulin signaling [36, 37]; the infiltrating macrophages are a major source of TNF*α* in the vascular wall [18]. Recently, we [38] have shown that genetic knockout of TNF*α* gene lowers blood pressure and vascular inflammation and protects against endothelial dysfunction and vascular injury in DOCA salt-sensitive hypertension. Here, we showed that TNF*α* expression was significantly increased in the vasculature of hypertensive DS rats; macrophage depletion reduced vascular TNF*α* expression and inhibited NF*κ*B and JNK activation associated with the improvement of vascular insulin signaling and insulin-mediated vasorelaxation. We have previously shown that inhibition of NF*κ*B by PDTC improves vascular insulin action via inhibition of TNF*α*/JNK activation in this animal model [17]. Therefore, we surmise that the improvement of vascular insulin action resulted from macrophage depletion may be attributed to the inhibition of the NF*κ*B/TNF*α*/JNK pathway. Macrophages are an important source of oxidative stress in the infiltrating tissue [39]; the depletion of macrophage significantly reduces vascular oxidative stress, which may partially help reduce cardiovascular injury.

In the present study, LEC significantly reduced SBP in hypertensive DS rats, which is consistent with previous reports showing that depletion of macrophage can lower blood pressure in hypertensive animals [10, 13]. Recently, we have shown that depletion of macrophage by LEC lowers blood pressure and improves renal function and injury in hypertensive animals [13]; therefore, the reduction in blood pressure after LEC treatment may be due to both the improvement of vascular and renal function and injury. It is well known that hemodynamic stress is an important factor to promote cardiovascular and renal injury in hypertension. Our previous studies in hypertensive DS rats showed that reduction in blood pressure by either thiazide diuretics or hydralazine slightly attenuated aortic and cardiac hypertrophy but did not provide other vascular beneficial effects, such as the protection against endothelial dysfunction or oxidative stress [40]. Therefore, reduction in blood pressure per se after macrophage depletion may to some extent help to reduce cardiac and vascular hypertrophy but may not help to improve endothelial insulin signaling and other vascular beneficial effects in this animal model.

## 5. Conclusions

Chronic low-grade inflammation is associated with hypertensive and metabolic diseases [41, 42]. The impairments of endothelial insulin NO production may importantly contribute to the development of vascular injury in hypertension, obesity, and type II diabetes [6, 43]. Our results suggest that in salt-sensitive hypertension, macrophages initiate vascular inflammation to induce vascular insulin resistance and cardiovascular injury, which may bridge risk factors for the development of cardiovascular and metabolic disorders [11, 12]. Macrophage may be a new target for immunotherapy of vasculopathy in hypertensive and metabolic diseases.

## Figures and Tables

**Table 1:** Body weight, aortic weight, heart rate, and *E*_max_ and ED50 value.

	NS	HS	HS/LEC
BW (g)	313 ± 8	295 ± 7	302 ± 7
AW/BW (mg/100 g BW)	10.8 ± 0.6	16.1 ± 0.4^∗^	13.8 ± 0.5^∗^^#^
Heart rate (bpm)	368 ± 21	402 ± 18	383 ± 25
Fasting glucose (mg/dL)	95 ± 5	101 ± 6	100 ± 7
KCl-induced contraction (g)	1.2 ± 0.15	1.1 ± 0.18	1.1 ± 0.12
*E*_max_ (relaxation, %)			
Acetylcholine	97 ± 2	68 ± 5^∗^	85 ± 4^∗^^#^
Insulin	30 ± 3	11 ± 3^∗^	23 ± 3^∗^^#^
ED50			
Acetylcholine	7.2 ± 0.1	6.8 ± 0.1^∗^	7.1 ± 0.1^#^
Insulin	7.5 ± 0.1	7.4 ± 0.2	7.4 ± 0.1

AW: aortic weight; BW: body weight; values are means ± SE; *n* = 8. Statistical analysis was performed by one-way ANOVA followed by the Bonferroni corrections for multiple comparisons between groups. ^∗^*p* < 0.05 vs. NS group; ^#^*p* < 0.05 vs. HS group.

## Data Availability

All data used to support the findings of this study are included within the article.
